# HormoneBase, a population-level database of steroid hormone levels across vertebrates

**DOI:** 10.1038/sdata.2018.97

**Published:** 2018-05-22

**Authors:** Maren N. Vitousek, Michele A. Johnson, Jeremy W. Donald, Clinton D. Francis, Matthew J. Fuxjager, Wolfgang Goymann, Michaela Hau, Jerry F. Husak, Bonnie K. Kircher, Rosemary Knapp, Lynn B. Martin, Eliot T. Miller, Laura A. Schoenle, Jennifer J. Uehling, Tony D. Williams

**Affiliations:** 1 Department of Ecology and Evolutionary Biology, Cornell University, Ithaca, NY 14853, USA; 2 Department of Biology, Trinity University, San Antonio, TX 78212, USA; 3 Coates Library, Trinity University, San Antonio, TX 78212, USA; 4 Biological Sciences Department, California Polytechnic State University, San Luis Obispo, CA 93407, USA; 5 Department of Biology, Wake Forest University, Winston-Salem, NC 27109, USA; 6 Max Planck Institute for Ornithology, Seewiesen 82319, Germany; 7 Department of Biology, University of St. Thomas, St. Paul, MN 55105, USA; 8 Department of Biology, University of Florida, Gainesville, FL 32608, USA; 9 Department of Biology, University of Oklahoma, Norman, OK 73019, USA; 10 Department of Global Health, University of South Florida, Tampa, FL 33620, USA; 11 Cornell Lab of Ornithology, Ithaca, NY 14850, USA; 12 Department of Biological Sciences, Simon Fraser University, Burnaby, BC V5A 1S6, Canada

**Keywords:** Animal behaviour, Ecophysiology, Physiology, Evolution, Animal physiology

## Abstract

Hormones are central regulators of organismal function and flexibility that mediate a diversity of phenotypic traits from early development through senescence. Yet despite these important roles, basic questions about how and why hormone systems vary within and across species remain unanswered. Here we describe HormoneBase, a database of circulating steroid hormone levels and their variation across vertebrates. This database aims to provide all available data on the mean, variation, and range of plasma glucocorticoids (both baseline and stress-induced) and androgens in free-living and un-manipulated adult vertebrates. HormoneBase (www.HormoneBase.org) currently includes >6,580 entries from 476 species, reported in 648 publications from 1967 to 2015, and unpublished datasets. Entries are associated with data on the species and population, sex, year and month of study, geographic coordinates, life history stage, method and latency of hormone sampling, and analysis technique. This novel resource could be used for analyses of the function and evolution of hormone systems, and the relationships between hormonal variation and a variety of processes including phenotypic variation, fitness, and species distributions.

## Background & Summary

Hormones are central regulators of phenotype, whose effects span multiple fields of research, from molecular biology to population biology^1–5^. Because of their role in regulating organismal function and flexibility, selection might be expected to constrain hormone levels or their context-dependent flexibility around one or more fitness optima^6–9^. Nevertheless, endocrine responses vary markedly both within and among populations^8,10–12^. Why do some individuals mount a hormonal response that is two or more orders of magnitude greater than others, when faced with the same stimulus? Similarly, why have some species evolved to express plasma testosterone levels that are an order of magnitude greater than others during reproduction, when testosterone mediates the same basic reproductive processes?

A particularly promising approach to answering such questions – and many others of broad interest to animal behaviour and organismal biology – lies in large-scale comparative analyses of the multitude of endocrine data that have been collected over the past several decades. Such analyses, conducted within a rigorous phylogenetic, environmental, and life-history framework, have the potential to illuminate the factors driving divergence in the hormonal mechanisms of behaviour, physiology, and morphology^13,14^. To date, most analyses have focused on relatively small taxonomic scales, and on comparing mean trait values across populations and species^15–19^. However, resources are rapidly becoming available to aggregate and analyse decades of available data on circulating hormone levels and their variation within free-living populations, across taxonomic groups. Identifying and characterizing the variation in endocrine traits, and their links with environment, life history, and fitness, could provide insight into how endocrine systems evolve, and how selection on these phenotypic integrators may influence the dynamics and distribution of populations^20–23^.

**Figure 1:**
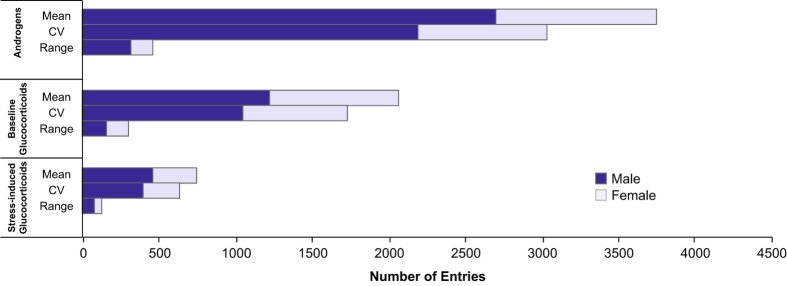
Total data entries in HormoneBase for each steroid by measurement type and sex. Within each category, counts are shown separately for mean, coefficient of variation, and range.

**Figure 2:**
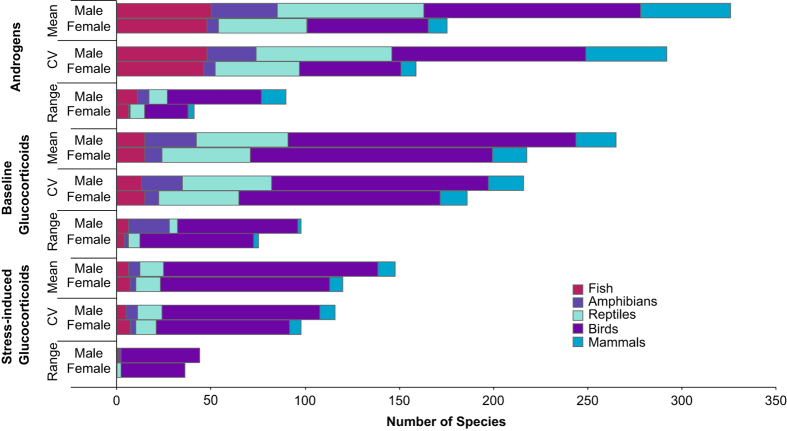
The number of species with data on mean hormone concentrations in HormoneBase. Counts are shown separately for males and females, and for androgens, baseline glucocorticoids, and stress-induced glucocorticoids.

**Figure 3:**
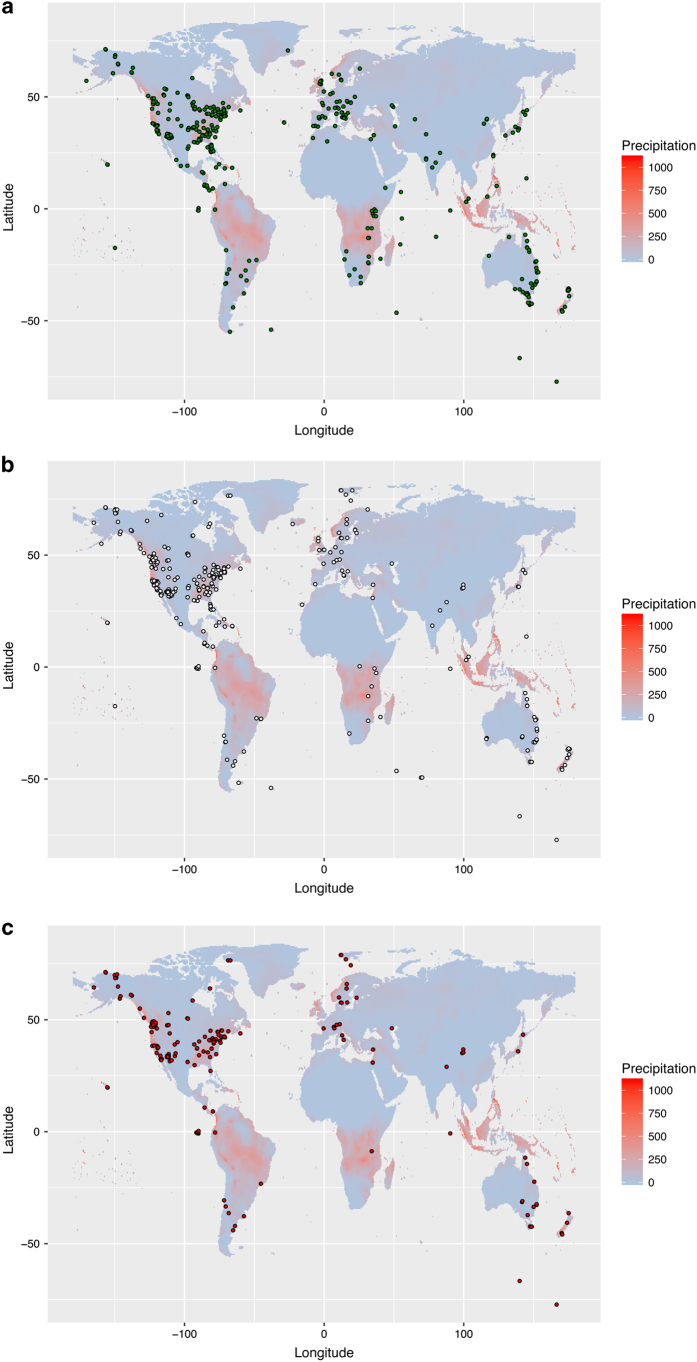
The geographical distribution of entries in HormoneBase. Points represent the location of measurements of (**a**) androgens, (**b**) baseline glucocorticoids, and (**c**) stress-induced glucocorticoids. Precipitation patterns reflect sums for December 2015 and were acquired from the CRU-TS 4.0 Climate Database^36^.

In this context, we present HormoneBase, a resource of compiled endocrine data across vertebrates. Included in this dataset are >6,580 measures of mean and within-population variation in glucocorticoids and androgens from 476 species (Figs 1,2; Table 1) that were reported in 648 publications – and additional unpublished resources – between 1967 and 2015. Additional information on geographic location (Fig. 3), life history, study design, and time period accompanies each entry. By making HormoneBase publicly available we aim to encourage data sharing across the scientific community and facilitate research into the function and evolution of physiological traits.

## Methods

### Hormonal Data

Endocrine data were obtained from publications, and from several unpublished datasets (Data Citation 1). We searched for studies that conformed to our inclusion criteria using: (i) online academic databases (e.g., Google Scholar, Web of Science), and (ii) cross-referencing from other published works. Studies were selected for inclusion if they included data on circulating glucocorticoids (baseline or stress-induced corticosterone/cortisol) or androgens (testosterone/11-ketotestosterone) that: (i) were from free-living populations, (ii) were collected from adults that had not been subject to an experimental manipulation prior to sampling (e.g., of hormones or the environment), (iii) measured plasma levels, (iv) did not pool data across males and females, or across adults and juveniles, and (v) were reported in or could be converted to a standard unit of measurement (ng/mL).

Published values were obtained from text, tables, or supplementary materials, or extracted from published figures using the program Data Thief III (http://datathief.org). Entries include mean circulating concentrations (ng/mL) for each population/group and time period; whenever possible, data on within-population variation (coefficient of variation, standard error), range (maximum and minimum values), and sample size are also included. When papers did not directly report the coefficient of variation (CV), it was calculated from the standard deviation (SD) or standard error (SE) and sample size (*n*), according to the following formulas: $CV=\frac{\mathrm{SD}}{\mathrm{mean}}*100$ or $\mathrm{CV}=\frac{\mathrm{SE}*\sqrt{n}}{\mathrm{mean}}*100$. If papers reported that outliers had been excluded we noted this for each hormone measure, and noted the criteria for exclusion where provided.

When a single reference reported multiple means for different groups of individuals (e.g., populations or life history stages), or from different time points, data were entered on separate lines. In cases where papers reported a single hormonal mean from data collected across multiple populations, the location of up to three of the sampled populations was noted in the entry. When stress-induced glucocorticoid levels were measured at multiple time points during a standardized stress series, only the time period at which mean glucocorticoid levels were highest was included.

The decision was made to focus HormoneBase on androgens and glucocorticoids because these are currently the most widely sampled hormones across vertebrates. Because hormone concentrations are not directly comparable across biological matrices, we included only plasma hormone concentrations. Hormone levels are also increasingly being measured in other biological matrices (e.g., feces, feathers) but these sample types are not very well-suited for large-scale comparative analyses because they use hormone metabolites, which differ within and across species and assay/antibody types^24^.

### Sample Collection and Assay Method Data

Because sampling method and assay technique may influence circulating hormone levels, we included specific information about capture, sampling, and assay approach. The time of day (i.e., range of hours) that samples were collected was recorded as provided. The specific method used to capture free-living individuals was noted, and the capture/sampling method assigned to one of three categories. “Active” sampling methods were those in which a blood sample was obtained rapidly and within a known period of time after targeting a previously undisturbed animal. “Passive” methods are those in which animals were sampled after an unknown period of restraint (e.g., non-continuously monitored traps or nets). “Attractant” methods are those in which animals were drawn to the site of capture using some type of attractant (e.g., song playbacks, baited traps). The maximum sampling latency (interval from capture to blood sampling) was recorded for androgens and baseline glucocorticoids, and the type of acute stressor and the interval from initial capture to the collection of a stress-induced glucocorticoid sample were also recorded.

To explore and control for potential differences in assay technique, we included information on the assay method used to assess plasma hormone levels (e.g., radioimmunoassay, enzyme immunoassay), and, where provided, the specific antibody or commercial kit that was used. Because laboratory identity can also influence measured hormone levels^25^, we recorded the identity of the laboratory in which hormone assays were conducted. For collaborative papers that did not identify where assays were conducted, and were the product of multiple endocrine laboratories, we arbitrarily assigned one of the collaborating laboratories as the assay location.

### Taxonomic and Geographical Data

All endocrine data include associated taxonomic information, using common and scientific names. Where relevant, scientific names were updated to reflect recent reclassifications. Taxonomy was determined using major lineage-specific trees (ray-finned fishes^26^, amphibians^27,28^, mammals^29^, squamates^30^, turtles^31^, and birds^32^).

The location name, geographic coordinates (latitude and longitude in degrees decimal), and elevation (in meters) of the population from which the data were collected are also recorded for each entry. When not provided in the original publication, approximate geographic coordinates and elevation were determined by searching for the location name in Google Earth.

### Temporal and Life History Data

To enable assessment of seasonal and life history patterns^17,33,34^, we included information on the time period of sampling as reported (the month and year in which data were collected) and the life history stage of sampled individuals. Measurements were characterized as coming from breeding or non-breeding individuals, or a combination of the two. Designations were based on author classifications when provided. When life history stage was not provided in the original data source, samples were classified as coming from a combination of breeding and non-breeding individuals, except in cases where seasonally breeding populations were sampled only during months that did not overlap with the breeding season.

When life history sub-stage was provided in the original data source, this information was also included in the database. To provide some standardization across species, and widely varying terminology, reported sub-stages were combined into fourteen categories: pre-breeding, courtship, incubation, copulation, gravid/pregnant, non-gravid/pregnant, laying, young care, lactation, post-breeding, migration, torpor, hibernation, pre-basic moult. When information about life history sub-stage was not contained in the original data source, this field was left blank. An associated column provides information about whether the sampled individuals were confirmed to be in a given life history stage (e.g., incubating birds captured off their nests), or whether the life history stage reflected the typical stage for individuals in that population at the time of sampling (e.g., birds sampled in mist nets during the breeding season but not traced to a specific nest). For birds, information on moult status was also recorded as provided^35^.

## Data Records

The HormoneBase dataset (Data Citation 1) is provided as two comma-separated values text files: a single file that includes all data described above (HormoneBase_v1.csv), and a file that contains the reference information for the source of each entry (HormoneBase_references_v1.csv). Variable names are provided in the first row, with details of each variable and units measured summarized in Table 2 (available online only). These files are accompanied by a metadata pdf file (HormoneBase_MetadataData.pdf).

## Technical Validation

The data presented in HormoneBase are primarily from published, peer-reviewed sources, but also contain unpublished data provided by authors. Data entry was initially proofed by each lab that entered the data to confirm that the entries matched reported data. Upon submission to the central repository, two members of the database entry team independently examined each entry to identify incomplete entries or extreme values. All hormone measures were also mapped onto a phylogeny to reveal putative taxonomic outliers. When such cases were identified, entries were confirmed or corrected by consulting the original source.

## Usage Notes

The data are available to access and download from *Figshare* repository (Data Citation 1). Three files are provided:

1. HormoneBase_v1.csv
2. HormoneBase_references_v1.csv
3. Metadata.pdf

## Additional information

**How to cite this article:** Vitousek, M. N. *et al.* HormoneBase, a population-level database of steroid hormone levels across vertebrates. *Sci. Data* 5:180097 doi: 10.1038/sdata.2018.97 (2018).

**Publisher’s note:** Springer Nature remains neutral with regard to jurisdictional claims in published maps and institutional affiliations.

## Supplementary Material



## Figures and Tables

**Table 1:** The taxonomic distribution of entries of mean circulating hormone levels in HormoneBase for males (M) and females (F).

	**Birds**		**Reptiles**		**Amphibians**		**Mammals**		**Fish**		**Total**	
	**M**	**F**	**M**	**F**	**M**	**F**	**M**	**F**	**M**	**F**	**M**	**F**
**Androgens**	1002	366	497	176	217	78	270	21	713	436	2699	1077
**Baseline GCs**	746	488	230	167	104	45	59	60	89	73	1228	833
**Stress-induced GCs**	352	212	26	28	29	7	20	27	23	20	450	294

**Table 2:** Variables included in HormoneBase

**Variable name**	**Variable definition**	**Data type/unit**
**Vert_Group**	Taxonomic group (amphibian, bird, fish, mammal, reptile)	String
**Genus**	Genus	String
**Species**	Species	String
**Common_name**	Common name	String
**Population_1**	Name of first location at which samples were collected (city/region, state/province, country)	String
**Population_2**	If applicable, name of second location at which samples were collected	String
**Population_3**	If applicable, name of third location at which samples were collected	String
**Latitude**	Latitude of primary sampling location	Degrees decimal
**Longitude**	Longitude of primary sampling location	Degrees decimal
**LatLongEst**	Were latitude and longitude reported in the paper or estimated by data entry team?	Binary (Reported/Estimated)
**Elevation**	Elevation at primary sampling location	Numerical (in m)
**Years**	Year(s) during which study was conducted	Year
**Year_1**	First year of study	Year
**Year_final**	Final year of study	Year
**Breeding_Cycle**	Data collected during breeding, non-breeding, or both	Breeding/Nonbreeding/Breeding, Nonbreeding
**Moult**	Measurements were collected during moult	Binary (Y/N)
**Life_Stage**	Life history sub-stage (when provided): Prebreeding, Courtship, Incubation, Copulation, Gravid/Pregnant, Non-Gravid/Pregnant, Laying, Young care, Lactation, Post-breeding, Migration, Torpor, Hibernation, Pre-basic moult. If hormones were measured for a distinct morph or social status, indicated here	String
**LifeHistConf**	Sampled individuals were confirmed to be in the designated life history stage	Binary (Y/N)
**Jan_Sampled**	Measures were collected in January	Binary (Y/N)
**Feb_Sampled**	Measures were collected in February	Binary (Y/N)
**Mar_Sampled**	Measures were collected in March	Binary (Y/N)
**Apr_Sampled**	Measures were collected in April	Binary (Y/N)
**May_Sampled**	Measures were collected in May	Binary (Y/N)
**June_Sampled**	Measures were collected in June	Binary (Y/N)
**July_Sampled**	Measures were collected in July	Binary (Y/N)
**Aug_Sampled**	Measures were collected in August	Binary (Y/N)
**Sept_Sampled**	Measures were collected in September	Binary (Y/N)
**Oct_Sampled**	Measures were collected in October	Binary (Y/N)
**Nov_Sampled**	Measures were collected in November	Binary (Y/N)
**Dec_Sampled**	Measures were collected in December	Binary (Y/N)
**Time_min**	Beginning of sampling window	Time (24h:min)
**Time_max**	End of sampling window	Time (24h:min)
**CaptureMethod**	Method of capture	String
**SampleMethod**	Sampling active (known time to sampling, no attractants), passive (captured for unknown period before sampling), or attractant (captured with aid of playback, baited traps, etc.)	Active, Passive, Attractant
**MaxLatency_A**	Maximum sampling latency for androgens	Numerical (min)
**MaxLatency_Cort**	Maximum sampling latency for baseline glucocorticoids	Numerical (min)
**LateStressCort**	Latency between capture and stress-induced sample	Numerical (min)
**StressorType**	Type of stressor applied (baseline to stress-induced)	String
**MajorStressPop**	Major stressor experienced by population (if relevant)	String
**Method**	Assay method	String
**A_AntibodyKit**	Kit or antibody used for androgen assays	String
**Cort_AntibodyKit**	Kit or antibody used for cort assays	String
**CORT**	Glucocorticoid measured	Binary (corticosterone/ cortisol)
**Androgen**	Androgen measured	Binary (testosterone/ 11-ketotestosterone)
**M_A_ Mean**	Males - mean androgen concentration	Numerical (ng/mL)
**M_A_SE**	Males - Standard error of androgens	Numerical
**M_A_CV**	Males - Coefficient of variation in androgens	Numerical
**M_A_N**	Males - Sample size for androgens	Numerical
**M_A_Min**	Males - Minimum reported androgen value	Numerical (ng/mL)
**M_A_Max**	Males - Maximum reported androgen value	Numerical (ng/mL)
**M_A_RemoveOutlier**	Males - Were androgen outliers removed?	Binary (Y/N)
**F_A_Mean**	Females - Mean androgen concentration	Numerical (ng/mL)
**F_A_SE**	Females - Standard error of androgens	Numerical
**F_A_CV**	Females - Coefficient of variation in androgens	Numerical
**F_A_N**	Females - Sample size for androgens	Numerical
**F_A_Min**	Females - Minimum reported androgen value	Numerical (ng/mL)
**F_A_Max**	Females - Maximum reported androgen value	Numerical (ng/mL)
**F_A_RemoveOutlier**	Females - Were androgen outliers removed?	Binary (Y/N)
**M_BC_Mean**	Males - mean baseline cort concentration	Numerical (ng/mL)
**M_BC_SE**	Males - Standard error of baseline cort	Numerical
**M_BC_CV**	Males - Coefficient of variation in baseline cort	Numerical
**M_BC_N**	Males - Sample size for baseline cort	Numerical
**M_BC_Min**	Males - Minimum reported value of baseline cort	Numerical (ng/mL)
**M_BC_Max**	Males - Maximum reported value of baseline cort	Numerical (ng/mL)
**M_BC_RemoveOutlier**	Males - Were baseline cort outliers removed?	Binary (Y/N)
**F_BC_Mean**	Females - Mean baseline cort concentration	Numerical (ng/mL)
**F_BC_SE**	Females - Standard error of baseline cort	Numerical
**F_BC_CV**	Females - Coefficient of variation in baseline cort	Numerical
**F_BC_N**	Females - Sample size for baseline cort	Numerical
**F_BC_Min**	Females - Minimum reported value of baseline cort	Numerical (ng/mL)
**F_BC_Max**	Females - Maximum reported value of baseline cort	Numerical (ng/mL)
**F_BC_RemoveOutlier**	Females – Were baseline cort outliers removed?	Binary (Y/N)
**M_SC_Mean**	Males - Mean stress-induced cort concentration	Numerical (ng/mL)
**M_SC_SE**	Males - Standard error of stress-induced cort	Numerical
**M_SC_CV**	Males - Coefficient of variation in stress-induced cort	Numerical
**M_SC_N**	Males - Sample size for stress-induced cort	Numerical
**M_SC_Min**	Males - Minimum reported value of stress-induced cort	Numerical (ng/mL)
**M_SC_Max**	Males - Maximum reported value of stress-induced cort	Numerical (ng/mL)
**M_SC_RemoveOutlier**	Males - Were stress-induced cort outliers removed?	Binary (Y/N)
**F_SC_Mean**	Females - Mean stress-induced cort concentration	Numerical (ng/mL)
**F_SC_SE**	Females - Standard error of stress-induced cort	Numerical
**F_SC_CV**	Females - Coefficient of variation in stress-induced cort	Numerical
**F_SC_N**	Females - Sample size for stress-induced cort	Numerical
**F_SC_Min**	Females - Minimum reported value of stress-induced cort	Numerical (ng/mL)
**F_SC_Max**	Females - Maximum reported value of stress-induced cort	Numerical (ng/mL)
**F_SC_RemoveOutlier**	Females – Were stress-induced cort outliers removed?	Binary (Y/N)
**OutlierCriteria**	Criteria for removing outliers	String
**Notes**	Notes on data	String
**Lab_ID**	PI of lab where assays conducted, if specified; if unspecified, arbitrarily assigned to one of the collaborating endocrine labs	String
**Ref_ID**	Reference to data source	Code
